# 

**Published:** 1916-11-25

**Authors:** 


### CONTENTS.

PAGE

**The Professional Fees of Physicians and Surgeons** ... 153

**Recent German Work on Tuberculosis** ... 154

**Hospital and Institutional News** ... 155

Dinner to Mr. Clive Bridgeman, M.P.—Lord Knutsford Prophetic—The Canadian Medical Service—A New Hospital Treasurer and his Record—The St. George's Hospital Extension—The Extension of the Special Board of Medical Appeal—A Medical Appeal Board for Manchester—A Pertinacious M.P. and his Memory—An Irish Wit on Annual Subscriptions—Ulster Strikers Stop a Hospital Building—The Health of Munition Workers—A Curious Suit Against the London Fever Hospital—Christmas at the Cardiff Military Hospitals—Sanatorium for Tuberculous London Children—A Point for Sanatorium Architects—The Shaftesbury Guardians and their Medical Officer—A New Institution for the Aged and for War Invalids at Kirkcaldy—A Remarkable Inquest Case—The War Opens the Way for Medical Women—Parliamentary Representation for Pharmacy—A West-Country Training School's Record—An Objectionable Circular—A Deplorable Conflict of Medical Evidence—The Fear of Hospitals—Hospitals and the Price of Newspapers—This Week's Drug Market—To Our Readers.

**Physical Treatment of War Injuries** ... 161

Zander Mechanotherapy.
Some Zander Machines Described and Illustrated.

PAGE

**A State Asylum Service** ... 165

A Change that would Promote Efficiency.

**Report of King Edward's Hospital Fund** ... 166

Statistics of 109 London Hospitals in 1915.

**Moral** ... 167

II.—Factors in its Destruction.

**Hospital and Institutional Results in 1915** ... 168

**Gifts to Hospitals** ... 168

**Passing Events and Topics** ... 168

**The Editor's Letter-Box** ... 169

Peter Bent Brigham Hospital—The College of Nursing, Limited, and State Registration of Nurses—Decorations for Hospital Secretaries—The West London Hospital—Sanatorium Buildings at £12 per Bed.

**The Matrons' and Sisters' Department** ... 171

The Training of Hospital Matrons.
Round the Hospitals.
The College and State Registration—Sir J. K. Fowler's Appointment—A Question of References—Regulating Off-Duty Hours—Winners of £50 Prize—Etiquette in Hospital Life.

**Matrons' and Sisters' Appointments** ... 172

**The Institutional Worker** ... (Suppl.) 1

War Conditions and Hospital Expenditure.
Question for November.
Enquiries and Answers.
The Sick and in Need.

